# Reevaluating manganese requirements of broiler chickens under phytase supplementation^☆^

**DOI:** 10.1016/j.psj.2026.107054

**Published:** 2026-05-01

**Authors:** W.E. Altevogt, S.L. Vieira, D.D.B. Maria, R.M. Horn, Y.J. Olabarriaga, L.A. Cunha, R.F.B. Leiskosky

**Affiliations:** Department of Animal Science, Federal University of Rio Grande do Sul, Av. Bento Gonçalves, 7712, Porto Alegre, RS 91540-000, Brazil

**Keywords:** Broiler, Manganese, Superoxide dismutase, Phytase

## Abstract

Manganese (Mn) is routinely supplemented in broiler diets to prevent deficiency and sustain optimal performance, while phytase is known to enhance trace mineral availability in corn–SBM feeds. This study evaluated graded Mn supplementation in diets with or without phytase. A total of 640 one-day-old male Cobb × Cobb 500 chicks were assigned to a 2 × 5 factorial design (with or without phytase × five Mn levels). Birds were housed in 80 battery cages, with 8 replicates of 8 birds each. From placement to 7 days, all chicks received a Mn-deficient diet without phytase (14.58 ± 0.74 mg/kg analyzed Mn). From 7 to 28 days, corn–SBM diets (19.64 ± 1.5 mg/kg analyzed Mn) were fed with phytase at 4,000 FYT/kg (analyzed 4,680 ± 420 FYT/kg) and Mn, supplied as MnSO₄·H₂O, to achieve 19.64 ± 1.5, 44.08 ± 0.67, 73.80 ± 1.26, 105.08 ± 2.76, and 129.97 ± 1.59 mg/kg analyzed Mn. No interactions between phytase and Mn were detected (P > 0.05). Phytase significantly improved growth performance, dry matter and Mn retention (P < 0.001), and AMEn (P < 0.05). Increasing dietary Mn had no effect on growth performance, locomotor parameters, or tibia morphometry at 28 days (P > 0.05), whereas Mn excretion and retention increased linearly with higher dietary inclusion (P < 0.001). Tissue Mn concentrations in tibia and liver, as well as heart Mn superoxide dismutase (Mn-SOD) activity, responded quadratically, with estimated maxima at 124.2, 180.6, and 105.8 mg/kg Mn, respectively (P < 0.05). These results indicate that current Mn supplementation practices in commercial broiler feeds may exceed requirements when growth performance is the primary response criterion. Improvements associated with phytase are consistent with previous findings for other trace minerals. However, Mn-SOD responses suggest that dietary Mn levels required to support antioxidant defense and mitigate oxidative stress may be higher than those needed to optimize conventional performance metrics.

## Introduction

Manganese (Mn) is essential playing important functions in animal metabolism. McHargue (1926) firstly demonstrated that Mn was essential for rats, whereas other authors have shown Mn deficiency in chicks (Wilgus et al., 1936) and pigs (Miller et al., 1940; Greenberg et al., 1943). The main functions of Mn are related to its role as a cofactor for several enzymes, including mitochondrial superoxide dismutase (MnSOD), pyruvate carboxylase, and arginase, which are involved in antioxidant defense, energy metabolism, and proteoglycan synthesis (Suttle, 2010; Tufarelli and Laudadio, 2017). Chicks store high amounts of Mn in the liver, kidneys and bones (Carvalho et al., 2021).

Manganese deficiency in broilers is primarily associated with skeletal abnormalities due to compromised activity of Mn-dependent enzymes, resulting in reduced proteoglycan synthesis and impaired cartilage and bone formation (Luo et al., 1992; Lu et al., 2006). This condition has been called perosis and is characterized by joint deformities and tendon displacements (Wilgus et al., 1936). Poor productive performance and impaired antioxidant systems have also been reported during Mn deficiencies (Lu et al., 2006; Carvalho et al., 2021). On the other hand, excessive Mn intake causes toxicity, reduced growth, and increases fecal Mn excretion, which eventually leads to an unnecessary increase in environmental Mn load (Oberleas, 1999; Tufarelli and Laudadio, 2017). Toxicity of Mn, however, occurs with abnormally high Mn feed contents at 1,500 mg/kg feed or higher (Miles et al., 2003; Sunder et al., 2006).

Present commercial recommendations for trace mineral supplementation in broilers vary widely. For instance, the NRC (1994); Rostagno et al. (2024); Cobb (2024), and Aviagen (2022) suggest 60, 70, 100, and 120 mg Mn/kg feed, respectively. Variable requirements are expected in nutritional studies due to experimental factors such as the evaluation criteria used, feed composition, and differences due to broiler genetics (Sakomura and Rostagno, 2007). However, Mn requirement studies that evaluate metabolic responses as well as growth in the presence of phytase are scarce with broiler chickens. Among the different sources of Mn available for poultry nutrition, Mn monohydrate sulfate (MnSO₄·H₂O) is widely used in commercial feeds due to its low cost and reasonable bioavailability (NRC, 1994).

Supplementation with phytase is widely adopted in animal feeds, mainly focusing on the increase in the availability of P and Ca bound to phytate. Recent studies, however, have shown that phytase can also increase the utilization of metal cations such as Fe (Feijo et al., 2023), Zn (Maria et al., 2024) and Cu (Soster et al., 2023). The wide disparity among current Mn supplementation recommendations, as well as the disregard of the positive effects of phytase on trace mineral availability, underscores the need for a reassessment of trace mineral requirements in phytase supplemented feeds. This is reinforced by the need to reduce production costs and mitigate unnecessary buildup of waste in the environment.

The objective of the present study was to evaluate the Mn requirements of broilers using corn-soy feeds and graded increases of Mn sulfate. Phytase was added in excess to the feeds such that phytate could be extensively and quickly degraded allowing for a maximum trace mineral release. The evaluations were conducted based on productive performance and other responses that are particularly sensitive to Mn.

## Material and methods

All procedures used in the present study were approved by the Ethics and Research Committee of the Federal University of Rio Grande do Sul, Porto Alegre.

### Bird husbandry and dietary treatments

A total of 640 1-d-old male Cobb × Cobb 500 chicks were randomly placed into 80 wire cages (0.9 × 0.4 m2). Each cage was equipped with trough feeders and drinkers (one per cage) and *ad libitum* access to water and mash feeds. Temperature at placement was 32°C, which was adjusted daily to maintain bird comfort throughout the study. Lighting was provided 24 h continuously throughout the first week followed by a 16:8 light: dark program to the end of the experiment. Chicks were given a common Mn-deficient feed (analyzed total 14.58 ± 0.74 mg/kg of Mn, Table 1) produced with corn, soybean meal (SBM), soybean protein concentrate (60% CP) and laboratory grade calcium carbonate and phosphoric acid from 1 to 7 d. At d 8 chicks were randomly allocated into a 2 × 5 factorial arrangement of feeds with or without phytase or having five graded increases of supplemental Mn. Each treatment was replicated 8 times, in a total of 10 treatments of 8 birds each. Feeds were formulated with energy and nutrients to optimize live performance as usual in commercial integrations. Diets contained 2,975 kcal/kg AME and 23% CP during the first week (depletion phase), and 3,000 kcal/kg AME, 22% CP, 1.10% Ca, 0.50% available P, and 0.41% digestible P during the experimental phase (Table 1).

**Table 1:** Ingredient and nutrient composition of feeds having supplemental phytase and graded increases of Mn.

Ingredient, kg/ton	Mn Deficient (1 to 7 d)	Experimental (8 to 28 d)
Corn 7.8%	65.29	51.97
Soybean meal 45%	5.38	37.68
Soybean protein concentrate 66%	23.20	-
Soybean oil	0.5	4.15
Ca carbonate^1^	2.70	2.15
Phosphoric acid 85%	1.50	1.34
Common salt	0.40	0.54
DL-Methionine, 99%	0.28	0.34
L-Lysine, 62,4%	0.15	0.24
L-Threonine, 98.5%	0.07	0.11
Choline chloride	0.28	0.23
Vitamin and mineral mix^2^	0.25	0.25
Celite^3^	-	1.00
Total	100.00	100.00

Calculated nutrient composition, % unless noted^4^		

AME_n_, kcal/kg	2,975	3,000
CP	23.0 (23.3 ± 0.78)	21.7 (22.02 ± 0.43)
Ca	1.10 (1.09 ± 0.02)	0.90 (1.02 ± 0.03)
Available P	0.50	0.45
Digestible P	0.44	0.41
Total P	0.63 (0.64 ± 0.03)	0.67 (0.68 ± 0.02)
Total phytate^5^	0.23	0.22
Na	0.18	0.23
Choline, mg/kg	2,500	2,500
Dig. Lys	1.24	1.22
Dig. TSAA	0.93	0.92
Dig. Thr	0.84	0.81
Dig. Trp	0.25	0.24
Dig. Arg	1.43	1.34
Dig. Val	0.94	0.91
Mn, mg/kg^6^	14.46 (14.58 ± 0.74)	17.48 (19.64 ± 1.5)
Phytase, FYT/kg^7^	-	4,000 (4,680 ± 420)

1 Calcium carbonate and phosphoric acid were laboratory-grade and guaranteed to not present significant amounts of other minerals.

2 Composition per kilogram of feed: vitamin A, 12,000 IU; vitamin D 3, 3,000 IU; vitamin C, 50 mg; vitamin E, 100 IU; vitamin K 3, 6 mg; vitamin B12, 35 µg; thiamin, 3 mg; riboflavin, 15 mg; vitamin B6, 6 mg; niacin, 40 mg; pantothenic acid, 25 mg; folic acid, 4 mg; biotin, 0.3 mg; Zn, 100 mg, Cu, 15 mg, Fe, 40 mg, Se, 0.45 mg and I, 2 mg.

3 Indigestible marker (Celite, Celite Corp., Lompoc, CA).

4 Values between parentheses were analyzed.

5 Phytate values ​​analyzed with NIRS, considering the levels of 0.18% for corn, 0.38% for soybean meal and 0.45% for soybean protein concentrate, adjusted proportionally to the composition of the diets.

6 Formulated Mn were 17.48, 47.48, 77.48, 107.48 and 137.48 mg/kg; analyzed Mn in the diets with and without phytase were 19.64 ± 1.5, 44.08 ± 0.67, 73.80 ± 1.26, 105.08 ± 2.76, 129.97 ± 1.59 mg/kg.

7 HiPhorius 40,000 FYT/g, Novozymes A/S, Bagsvaerd, Denmark (not given nutrient matrix values).

Diets were formulated to minimize total Mn content while maintaining nutrient specifications consistent with commercial feeds during the first week. From 8 to 28 d, Mn in the experimental diets was derived solely from corn and soybean meal.

A commercial phytase (HiPhorius; 40,000 FYT/g; Novozymes A/S, Bagsvaerd, Denmark) was included at 100 g/ton of feed to provide 4,000 FYT/kg (analyzed 4,680 ± 420 FYT/kg) in half of the experimental diets. The applied dose was above standard commercial recommendations (1,000 FYT/kg) to facilitate rapid and extensive phytate degradation in corn–soybean meal–based diets. Phytase was added without assigning matrix credits for Ca or P and was incorporated through 1 kg premixes diluted in ground soybean meal.

Laboratory-grade Mn sulfate monohydrate (MnSO₄·H₂O) was added to the diets to achieve supplemental levels of 0, 30, 60, 90, and 120 mg Mn/kg. As in the depletion diet, calcium carbonate and phosphoric acid were of laboratory grade to minimize Mn contribution.

Analyzed phytase activity in the experimental diets ranged from 4,015 to 4,457 FYT/kg across Mn inclusion levels. Analyzed Mn concentrations in diets with and without phytase were 19.64 ± 1.5, 44.08 ± 0.67, 73.80 ± 1.26, 105.08 ± 2.76, and 129.97 ± 1.59 mg/kg. Celite (Celite Corp., Lompoc, CA) was included at 1% as an indigestible marker. Diets had an average geometric mean diameter of 1.118 μm ± 1.72. Phytase activity was expressed in FYT units, corresponding to the release of 1 μmol of inorganic phosphate per minute from 5.0 mM sodium phytate at pH 5.5 and 37°C (Engelen et al., 1994).

### Growth performance, total excreta, ileal contents

Live performance was evaluated based on body weight gain (BWG), feed intake (FI), and feed conversion ratio (FCR), corrected for the weight of dead birds, at 8, 14, 21, and 28 d of age. Excreta were collected twice daily from 24 to 27 d on wax paper, immediately pooled by cage, homogenized, and stored at −20°C until analysis. At 28 d of age, birds were euthanized by electrical stunning (45 V for 3 s), and ileal digesta were collected from the intestinal segment between Meckel’s diverticulum and approximately 2 cm proximal to the ileocecal junction. The contents were gently flushed with distilled water into plastic containers, pooled by cage, rapidly frozen in liquid nitrogen, and stored at −20°C until analysis. Samples were then lyophilized (Christ Alpha 2-4 LD Freeze Dryer, Newtown, UK), and both feed and dried ileal digesta were ground to pass a 0.5-mm sieve using a laboratory mill (Tecnal TE-631/2, São Paulo, Brazil).

### Analyses and calculations

Ileal digesta, excreta, and feed samples were analyzed for gross energy (GE) using a calorimeter calibrated with benzoic acid as a standard (IKA Werke, Parr Instruments, Staufen, Germany). Calculations of ileal digestible energy (IDE) and AME were done afterwards. Crude protein (CP, N × 6.25) was determined by combustion (method 968.06; AOAC International, 2016). Acid insoluble ash, ileum samples, and excreta were determined as described by Vogtmann et al. (1975) and Choct and Annison (1992). Apparent ileal digestibility, total tract retention, IDE, and AME were calculated using the equations of Kong and Adeola (2014) as: digestibility (%) = [1 − (Mi /Mo) × (Eo /Ei)] × 100, IDE and AME (kcal/kg) = GEi − [GE × (Mi /Mo)], where Mi represents the concentration of acid insoluble ash in the diet in grams per kilogram of DM; Mo represents the concentration of acid insoluble ash in the excreta and ileal digesta in grams per kilogram of DM output; Ei represents the concentration of DM, PB, GE, Mn in the diet in milligrams per kilogram of DM; and Eo represents the concentration of DM, PB, GE, Mn in the excreta and ileal digesta in milligrams per kilogram of DM. Retention of Mn was determined as follows: Mn retention (mg/bird) = [feed intake (g/bird) × feed Mn content (mg/kg)] − [fecal output (g/bird) × fecal Mn content (mg/kg)]. The diet and fecal Mn contents were based on their analyzed values on DM. All analyzes of Mn in the present research were conducted using Inductively Coupled Plasma Atomic Emission Spectrometry, (5800 ICP-OES, Agilent Technologies, Santa Clara, CA, USA), method of Association of Official Analytical Chemists (method 968.08; AOAC, 2016).

### Locomotor parameters and Tibia evaluation

At 14 and 28 d, valgus, varus, and rotated tibiae deviations at the tibia metatarsal joints were evaluated in all birds standing in anatomic position and evaluated by a 3 individual panel, classifying them by the presence or the absence of each deviation. Tibiae-metatarsal joints with valgus deviations were those presenting an outward angulation, whereas the varus deviations were inward. Rotated tibiae above 90° were considered abnormal and characterized as a torsional rotation of the shaft of the tibiotarsus of 1 or both legs, causing the metatarsus to point laterally and a spraddle leg conformation. Scores taken were averaged for the sake of statistical analysis as described by Santiago et al. (2020).

Gait score assessments were performed on d 14 and 28 of the experiment, following the methodology described by Garner et al. (2002), which classifies broiler locomotion on a scale from 0 (normal walking) to 5 (unable to walk). Three trained evaluators, blinded to treatment groups, independently scored the locomotion of chickens through a linear path of one meter.

The left tibiae of all birds from each cage were collected and the surrounding sampled muscle tissue removed and frozen until analysis. Tibia breaking strength was evaluated using a texture analyzer (TA.XTPlus, Texture Technologies, Hamilton, MA, United States), according to the method proposed by Shim et al. (2012). Tibia morphometry was performed with a micrometer (Model IP65, Mitutoyo Corp., Kawasaki, Japan) measuring length, diaphysis, caudal zone, and cranial region, and these values were calculated for statistical analysis (Maria, 2024). The tibiae were then defatted and ashed for Mn content analysis.

### Tissue sampling and analysis

Livers from all birds were collected at 28 d and immediately weighed being stored in plastic bags per battery cage at -20°C. Liver samples were lyophilized (Christ Alpha 1-8 LD Freeze Dryer, Newtown, UK), subsequently ashed, and analyzed for Mn content. Additional liver samples were defatted with ethyl ether for determination of fat content.

Heart samples were homogenized by sonication in PBS buffer solution (pH 7.4), and then the supernatant was collected after centrifugation at 4,000 rpm for 3 min at 4°C. Activity of MnSOD was measured in the supernatant using a Chicken Superoxide Dismutase 2, Mitochondrial (SOD2) ELISA Kit (Ref. MBS742418, My Bio Source, San Diego, CA, USA). Immediately, the protein concentration in the supernatant was determined by the Bradford method using BSA as a standard. MnSOD activity was expressed in ng/mg of protein.

### Statistical analysis

Data were tested for homoscedasticity and normality of the variance prior to statistical analyses (Shapiro and Wilk, 1965). Data without normal distribution was analyzed after arc-sin transformation, but the actual means are presented in the tables. A two-way ANOVA was used to test for differences in the effects of the independent variables (phytase and dietary Mn) on the dependent variables measured. Analyses were done using the GLM procedure of SAS (SAS, 2012) with significance accepted as P ≤ 0.05. Mean separation was done using Tukey multiple-range test when the model effect was significant (Tukey, 1991). Estimations of maximum responses to total dietary Mn were done using linear and quadratic regression models.

## Results and discussion

The formulated feeds were accepted as adequate for the experimental purposes since analyzed phytase and Mn in feeds were very close to the expected formulations. Phytase activity averaged 4,680 ± 420 FYT/kg, whereas analyzed Mn concentrations increased as formulated, reaching 19.64 ± 1.5, 44.08 ± 0.67, 73.80 ± 1.26, 105.08 ± 2.76, and 129.97 ± 1.59 mg/kg (Table 1).

There were no interactions between phytase and Mn for any of the responses evaluated throughout the present study; therefore, results and discussion are presented based on main effects. Lack of interaction effects of phytase on the dietary levels of metal cations has been previously demonstrated with Cu (Soster et al., 2023), Fe (Feijo et al., 2023) and Zn (Maria et al., 2024).

In the present study, phytase supplementation led to significant improvements in BWG and FCR between 8 and 28 d of age (*P* < 0.05) (Table 2). These positive effects reinforce the importance of phytase in the degradation of phytate, a compound that has been shown to restrict trace mineral availability (Soster et al., 2023; Feijo et al., 2023; Maria et al., 2024). Improvements in Ca and P availability, however, were not expected in the present study since phytase was added on top of the feeds without considering matrix values for these nutrients in feed formulation. On the other hand, increased dietary Mn did not lead to any significant responses in live performance (P > 0.05).

**Table 2:** Growth performance of broilers fed diets with supplemental phytase and graded increases of Mn from 8 to 28d^1^^,^^2^.

Item	BWG, g				FCR				FI, g			
	8 – 14	15 – 21	22 – 28	8 - 28	8 – 14	15 – 21	22 – 28	8 - 28	8 – 14	15 – 21	22 – 28	8 - 28
Phytase, FYT/kg^3^												
None	304	549^b^	693	1,548	1.286	1.300	1.390	1.335	391	717	970	2,083
4,000	310	566^a^	713	1,600	1.265	1.283	1.354	1.316	393	727	962	2,097
Mn, mg/kg^4^												
17.5	311	548	701	1,561	1.263	1.290	1.373	1.326	393	712	964	2,081
47.5	306	554	697	1,557	1.292	1.292	1.392	1.329	392	718	965	2,105
77.5	304	566	701	1,579	1.287	1.302	1.379	1.338	392	737	969	2,112
107.5	307	557	701	1,566	1.263	1.289	1.348	1.312	389	715	964	2,065
137.5	307	569	714	1,594	1.270	1.283	1.376	1.323	394	727	978	2,086
SEM	1.561	2.857	4.090	5.283	0.006	0.006	0.007	0.004	1.709	4.717	5.543	8.119
*Probability*												
Phytase	0.024	0.004	0.013	0.001	0.045	0.016	0.006	0.003	0.588	0.080	0.425	0.434
Mn	0.332	0.061	0.685	0.093	0.242	0.565	0.243	0.155	0.879	0.058	0.671	0.286
Phytase*Mn	0.066	0.203	0.659	0.960	0.735	0.055	0.078	0.236	0.302	0.105	0.143	0.085

a,b Means with different letters in the same column indicate significant differences (*P* ≤ 0.05).

1 BWG, body weight gain; FCR, feed conversion ratio corrected for the weight of dead birds; FI, feed intake.

2 Chick body weight at 1 and 8 d, respectively: 51.0 g ± 0.99; 207.6 g ± 4.67.

3 HiPhorius 40,000 FYT/g, Novozymes A/S, Bagsvaerd, Denmark.

4 Formulated Mn: 17.48, 47.48, 77.48, 107.48 and 137.48 mg/kg; analyzed Mn in feeds with and without phytase: 19.64 ± 1.5, 44.08 ± 0.67, 73.80 ± 1.26, 105.08 ± 2.76, 129.97 ± 1.59 mg/kg.

Chicks fed increased dietary Mn did not show significant differences in live performance (P > 0.05); however, contents of Mn in liver and tibiae increased as dietary Mn was gradually added to the feeds (*P* < 0.05). Quadratic adjustments were detected for tibia and liver Mn content at 28 d (Y = 2.5712 + 0.04488x - 0.000180708x²; R² = 0.1615; P < 0.0004, and Y = 5.1833 + 0.02013x - 0.000055722x²; R² = 0.773; P < 0.0001) (Table 5, Table 7). Maximum responses were at 124.2 mg (Table 5) and 180.6 mg Mn/kg of diet (Table 7), respectively for tibiae and liver contents. Previous research has not shown live performance improvements when Mn was supplemented in commercial type corn–SBM feeds for broilers indicating that they provide sufficient Mn to sustain maximum growth (Wedekind et al., 1992; Lu et al., 2006). Since the increased Mn concentrations in bone and liver tissues were not accompanied by improvements in growth performance, it seems that they do not appear to reflect an increased metabolic demand for Mn. Instead, these responses likely represent greater Mn deposition as dietary intake increases, consistent with patterns reported for other trace minerals (Bao et al., 2007). Additionally, higher liver Mn concentrations are expected with increased intake, as the liver plays a central role in Mn homeostasis and biliary excretion (Bao and Choct, 2009).

Incidence of leg deformities (Table 3), and other parameters, such as gait scores (Table 4), bone strength, and tibia morphometry (Table 5) were not affected by phytase and Mn in the present study. This is an indication that Mn, although essential for enzymes related to bone matrix formation, was provided by sufficient amounts from corn and SBM. Similarly, Li et al. (2004) reported that Mn supplementation, at relatively levels above requirements, did not affect leg deformities. It should be noted that birds in the present study were reared in battery cages and only to 28 d, which imposes limitations on the interpretation of the results. Increased body weight in broilers has been associated with a higher incidence of locomotor disorders due to greater skeletal loading as birds age (Williams et al., 2000; Knowles et al., 2008). Therefore, extrapolation of locomotor responses to heavier body weights at later processing ages may not be supported by the present findings.

**Table 3:** Leg deviations of broilers fed diets with phytase and graded increases of Mn from 8 to 28 d^1^^,^^2^.

Item	Leg deviations 14 d, %				Leg deviations 28 d, %			
	Unaffected	Valgus	Varus	Rotated tibia	Unaffected	Valgus	Varus	Rotated tibia
Phytase, FYT/kg^3^								
None	89.8	5.51	4.69	-	80.9	10.7	7.7	0.70
4,000	87.2	5.54	7.26	-	84.1	10.3	5.2	0.40
Mn, mg/kg^4^								
17.5	85.4	7.81	6.79	-	81.5	9.53	7.76	1.21
47.5	87.5	5.73	6.77	-	79.5	11.5	8.43	0.57
77.5	93.9	3.33	2.77	-	82.7	10.1	7.20	0.00
107.5	88.9	4.99	6.11	-	89.4	6.81	3.79	0.00
137.5	87.0	5.72	7.28	-	80.9	13.9	4.57	0.63
SEM	1.180	0.869	0.898	-	1.219	1.034	1.013	0.223
*Probability*								
Phytase	0.299	0.991	0.179	-	0.195	0.824	0.319	1.000
Mn	0.226	0.653	0.508	-	0.081	0.309	0.549	0.449
Phytase*Mn	0.802	0.499	0.418	-	0.244	0.101	0.661	0.595

1 Unaffected had no visible deviation in leg conformation, valgus had an outward angulation, varus had an inward angulation, and rotated tibia had abnormal internal or external rotation of the tibia relative to the longitudinal axis of the leg.

2 Data were arcsine transformed prior to analysis, but the values in the table are real means.

3 HiPhorius 40,000 FYT/g, Novozymes A/S, Bagsvaerd, Denmark.

4 Formulated Mn: 17.48, 47.48, 77.48, 107.48 and 137.48 mg/kg; analyzed Mn in feeds with and without phytase: 19.64 ± 1.5, 44.08 ± 0.67, 73.80 ± 1.26, 105.08 ± 2.76, 129.97 ± 1.59 mg/kg.

**Table 4:** Gait scores of broilers fed diets with phytase and graded increases of Mn from 8 to 28d, % of birds within each score^1^^,^^2^.

Item	Gait score 14 d						Gait score 28 d					
	0	1	2	3	4	5	0	1	2	3	4	5
Phytase, FTY/kg^3^												
None	85.0	13.3	1.70	-	-	-	61.9	29.9	7.10	1.10^(1.^^1^^)^	-	-
4,000	83.3	15.0	1.70	-	-	-	57.1	32.2	9.00	1.70^(1.5)^	-	-
Mn, mg/kg^4^												
17.5	80.7	18.2	1.10	-	-	-	60.9	30.2	7.80	1.11	-	-
47.5	84.9	13.0	2.10	-	-	-	57.9	28.9	11.60	2.10	-	-
77.5	87.8	11.1	1.10	-	-	-	59.2	32.5	8.30	0.60	-	-
107.5	86.5	10.9	2.60	-	-	-	61.0	33.8	5.20	0.50	-	-
137.5	81.3	17.2	1.50	-	-	-	58.9	29.7	7.29	2.70	-	-
SEM	1.225	1.138	0.378	-	-	-	1.552	1.408	0.919	0.431	-	-
*Probability*												
Phytase	0.521	0.491	0.970	-	-	-	0.172	0.428	0.317	0.232	-	-
Mn	0.309	0.144	0.689	-	-	-	0.971	0.798	0.326	0.368	-	-
Phytase*Mn	0.867	0.872	0.949	-	-	-	0.775	0.447	0.817	0.785	-	-

1 Locomotion scores ranged from 0 to 5 indicating: 0-skillful and agile movement, 1-slight and vague abnormality, 2-defined and identifiable abnormality, 3-obvious gait issue affecting mobility, 4-severe abnormality with limited movement, and 5-birds unable to move (Garner et al., 2002).

2 Data were arcsine transformed prior to analysis, but the values in the table are the real means.

3 HiPhorius 40,000 FYT/g, Novozymes A/S, Bagsvaerd, Denmark.

4 Formulated Mn: 17.48, 47.48, 77.48, 107.48 and 137.48 mg/kg; analyzed Mn in feeds with and without phytase: 19.64 ± 1.5, 44.08 ± 0.67, 73.80 ± 1.26, 105.08 ± 2.76, 129.97 ± 1.59 mg/kg.

**Table 5:** Proximal analyses, Mn content and tibia morphometry of broiler chickens at 28 d of age.

Item	Weight	DM	Ash	Mn	Breaking strength	Length	Proximal Epiphysis	Diaphysis	Distal Epiphysis
	g	%		mg/kg^2^	kgf	cm			
Phytase, FYT/kg^1^									
None	8.82	45.1	21.7	4.87	51.9	7.74	2.09	0.84	1.67
4,000	8.93	45.0	22.0	4.41	50.0	7.67	2.09	0.86	1.68
Mn, mg/kg^3^									
17.5	8.97	44.8	21.6	3.37^b^	51.0	7.68	2.12	0.86	1.69
47.5	8.68	45.4	22.0	4.40^ab^	49.7	7.74	2.05	0.83	1.64
77.5	8.81	45.1	21.8	4.86^ab^	48.1	7.69	2.08	0.86	1.69
107.5	8.95	45.1	22.0	5.13^a^	57.1	7.75	2.11	0.84	1.69
137.5	8.96	45.0	21.8	5.44^a^	52.3	7.67	2.08	0.84	1.67
SEM	0.082	0.129	0.105	0.211	0.765	0.238	0.092	0.061	0.087
*Probability*									
Phytase	0.491	0.737	0.150	0.238	0.242	0.183	0.637	0.141	0.576
Mn	0.752	0.599	0.655	0.016	0.067	0.827	0.156	0.328	0.164
Phytase*Mn	0.208	0.489	0.168	0.560	0.143	0.278	0.524	0.462	0.269

1 HiPhorius 40,000 FYT/g, Novozymes A/S, Bagsvaerd, Denmark. ^2^Tibia Mn content at 28d: Y = 2.5712 + 0.04488x -0.000180708x^2^, R^2^ = 0.1615, *P* = 0.0004; X = dietary Mn content.

3 Formulated Mn: 17.48, 47.48, 77.48, 107.48 and 137.48 mg/kg; analyzed Mn in feeds with and without phytase: 19.64 ± 1.5, 44.08 ± 0.67, 73.80 ± 1.26, 105.08 ± 2.76, 129.97 ± 1.59 mg/kg.

Total DM retention and AME_n_ were higher in birds fed supplemental phytase, confirming the positive effects of the enzyme on nutrient and energy utilization efficiency. The improvements in growth performance may reflect the observed benefits in energy utilization. Phytase also led to increased Mn retention, which was likely originated by the increased availability of Mn bound to phytate complexes. Similar results were reported by Li et al. and Akter et al. (2017), who observed greater Mn availability and tissue deposition in broilers fed diets supplemented with phytase.

Excretion and numerical retention of Mn occurred in parallel with dietary intake with (respective adjustments were Y = 0.8223 + 0.0313x, R^2^ = 0.666, *P* < 0.001; Y = -0.8004 + 0.0966x, R^2^ = 0.9177, *P* < 0.001). Percent Mn retained, however, decreased as Mn intake increased, indicating lower efficiency as Mn becomes dietary excessive (Table 6). This pattern has been shown in previous studies indicating that Mn absorption in broilers is tightly controlled, with excess being rapidly excreted through bile and then excreta (Tufarelli and Laudadio, 2017; Li et al., 2008).

**Table 6:** Apparent ileal digestibility and total tract retention of broilers as affected by increased dietary Mn with or without phytase, DM basis^1^^,^^2^.

Item	Apparent ileal digestibility					Total tract retention		Intake	Excretion	Retention	
	DM, %	IDEC, %	IDE, kcal/kg	CP, %	Mn, %	DM, %	AME, kcal/kg	Mn, mg/bird		Mn, mg/bird	Mn, %
Phytase, FYT/kg^3^											
None	75.4	75.7	3,431	83.8	17.9	76.7	3,600	9.7	4.3^a^	5.3^a^	55.5^a^
4,000	75.6	75.9	3,485	84.1	18.2	79.0	3,642	9.5	3.6^b^	6.0^b^	63.7^b^
Mn, mg/kg^4^											
17.5	76.2	76.6	3,450	83.9	16.3	77.9	3,610	2.5^e^	1.0^e^	1.5^e^	60.9
47.5	76.3	75.1	3,461	84.9	17.5	77.3	3,620	5.6^d^	2.2^d^	3.4^d^	60.8
77.5	74.2	74.3	3,383	83.1	17.7	76.0	3,608	9.6^c^	3.8^c^	5.8^c^	60.1
107.5	74.5	76.5	3,475	83.3	19.4	77.6	3,630	13.3^b^	5.5^b^	7.7^b^	58.5
137.5	76.9	76.8	3,534	85.4	17.8	78.1	3,654	16.9^a^	7.1^a^	9.8^a^	57.9
SEM	0.366	0.713	18.580	0.288	0.327	0.530	10.831	0.594	0.348	0.263	0.563
*Probability*											
Phytase	0.457	0.571	0.628	0.978	0.838	0.034	0.0252	0.6476	<0.0001	<0.0010	<0.0001
Mn	0.174	0.785	0.304	0.146	0.958	0.163	0.4738	<0.0001	<0.0001	<0.0001	0.1766
Phytase*Mn	0.433	0.673	0.074	0.350	0.742	0.510	0.4452	0.4200	0.4878	0.1650	0.7835

a>b Means with different letters in the same column indicate significant differences (P < 0.05).

1 IDCE = Ileal digestible energy coefficient; IDE = Ileal digestible energy.

2 Intake, excretion and retention, of Mn (mg/kg), respectively: Y = 0.0226 + 0.1279x, R^2^ = 0.9552, *P* < 0.001; Y = 0.8223 + 0.0313x, R^2^ = 0.666, *P* < 0.001; Y = -0.8004 + 0.0966x, R^2^ = 0.9177, *P* < 0.001; X = dietary Mn content.

3 HiPhorius 40,000 FYT/g, Novozymes A/S, Bagsvaerd, Denmark; analyzed phytase in the Mn supplemented diets were (from the lowest to the highest Mn content diets) 4,680 ± 420 FYT/kg.

4 Formulated Mn: 17.48, 47.48, 77.48, 107.48 and 137.48 mg/kg; analyzed Mn in feeds with and without phytase: 19.64 ± 1.5, 44.08 ± 0.67, 73.80 ± 1.26, 105.08 ± 2.76, 129.97 ± 1.59 mg/kg.

Manganese is a critical cofactor for MnSOD, and tissue Mn levels are closely associated with mitochondrial antioxidant capacity rather than productive performance (Surai, 2015). It is a specific and sensitive biomarker for circulating Mn without being directly influenced by other minerals within the same metabolic pathway and particularly pronounced in tissues with a high mitochondrial density, such as the heart (Li et al., 2008). In the present study, a quadratic increase in heart MnSOD activity occurred as a function of dietary Mn (MnSOD = Y = 10.196492 + 0.314327x −0.001486×2, R2 = 0.1377, P < 0.0183) (Table 7). The results show that cardiac MnSOD sensitivity to dietary Mn had a maximum response at 105.8 mg Mn/kg of feed. This behavior is consistent with the physiological mechanism of the enzyme, which depends directly on the incorporation of Mn as cofactor. Therefore, in contrast to the lack of response for growth and locomotor responses, increased dietary Mn seem to be needed to support heart’s mitochondrial antioxidant protection. The commercial significance of this impact, however, could not be detected in the present study.

**Table 7:** Concentration of Mn in liver and MnSOD activity in heart tissue at 28 d^1^.

Item	Liver			Heart	
	Weight, g	Mn, mg/kg	Fat, %	Weight, g	MnSOD, ng/mg protein
Phytase, FYT/kg^2^					
None	39.2	7.12	9.12	10.5	23.1
4,000	38.9	6.96	8.61	10.2	23.1
Mn, mg/kg^3^					
17.5	40.4	5.59^c^	9.69	9.97	13.5^b^
47.5	39.0	6.21^bc^	8.02	10.6	24.9^ab^
77.5	39.1	6.91^b^	9.58	10.3	23.1^ab^
107.5	38.5	7.97^a^	8.39	10.4	24.8^ab^
137.5	38.5	8.72^a^	8.23	10.5	26.7^a^
SEM	0.343	0.206	0.405	0.121	0.582
*Probability*					
Phytase	0.707	0.723	0.357	0.305	0.844
Mn	0.419	<0.0001	0.473	0.491	0.036
Phytase*Mn	0.465	0.947	0.169	0.313	0.711

1 Liver Mn content at 28 d: Y = 5.18328 + 0.02013x - 0.000055722x^2^, R^2^ = 0.773, *P* < 0.0001; MnSOD in heart content at 28 d: Y = 10.19649230 + 0.31432684x −0.00148597×2, R2 = 0.1377, P < 0.0183; X = dietary Mn content.

2 HiPhorius 40,000 FYT/g, Novozymes A/S, Bagsvaerd, Denmark.

3 Formulated Mn: 17.48, 47.48, 77.48, 107.48 and 137.48 mg/kg; analyzed Mn in feeds with and without phytase: 19.64 ± 1.5, 44.08 ± 0.67, 73.80 ± 1.26, 105.08 ± 2.76, 129.97 ± 1.59 mg/kg.

Results of the present study indicate that Mn supplementation of corn–soybean meal diets does not improve productive performance or skeletal health in broiler chickens. However, MnSOD activity in the heart responded positively to increasing dietary Mn, suggesting that enzymatic and metabolic functions may be more sensitive indicators of Mn adequacy than traditional performance traits. Considering the low retention efficiency observed at higher Mn intake levels, supplementation strategies should aim to balance physiological requirements with excesses that do not translate into productive benefits and may contribute to environmental accumulation. These findings highlight the importance of incorporating MnSOD and other Mn-dependent enzymatic responses into the evaluation of Mn requirements. Further research should investigate these biomarkers across multiple tissues to support more precise dietary recommendations.

## CRediT authorship contribution statement

**W.E. Altevogt:** Writing – review & editing, Writing – original draft, Supervision, Project administration, Methodology, Investigation, Formal analysis, Data curation. **S.L. Vieira:** Writing – review & editing, Visualization, Supervision, Resources, Funding acquisition, Conceptualization. **D.D.B. Maria:** Writing – review & editing, Validation, Investigation, Formal analysis. **R.M. Horn:** Writing – review & editing, Validation, Investigation, Formal analysis. **Y.J. Olabarriaga:** Writing – review & editing, Validation, Investigation, Formal analysis. **L.A. Cunha:** Writing – review & editing, Validation, Investigation, Formal analysis. **R.F.B. Leiskosky:** Investigation.

## Disclosures

All authors declare that they have no conflict of interest or personal relationships that could have appeared to influence the work reported in this article.
